# *QuickStats:* Percentage* of Adults Aged ≥18 Years who Received an Influenza Vaccination in the Past 12 Months,^†^ by Race and Ethnicity^§^ and Family Income^¶^ — National Health Interview Survey, United States, 2021

**DOI:** 10.15585/mmwr.mm7230a7

**Published:** 2023-07-28

**Figure:**
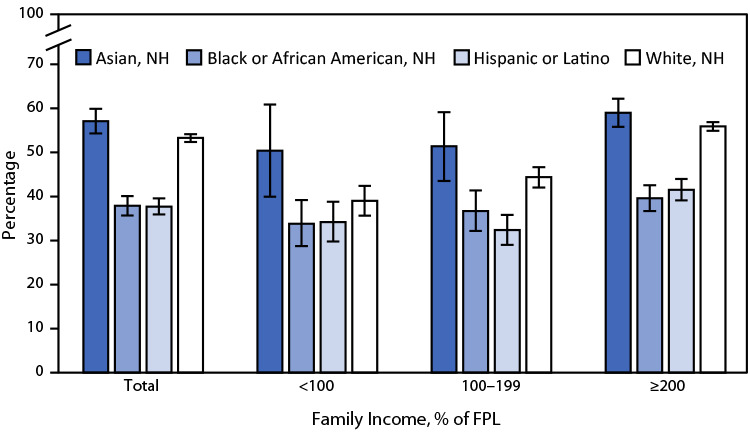
In 2021, non-Hispanic Asian (Asian) adults aged ≥18 years were the most likely to receive an influenza vaccination in the past 12 months (57.1%) followed by non-Hispanic White (White) (53.3%) adults; Hispanic or Latino (Hispanic) and non-Hispanic Black or African American (Black) adults were the least likely to receive an influenza vaccination (37.7% and 37.9%, respectively). Among adults with family incomes 100%–199% and ≥200% of FPL, Hispanic and Black adults were significantly less likely than Asian and White adults were to receive an influenza vaccination. Among adults with family incomes <100% of FPL, the differences among Hispanic, Black, and White adults were not statistically significant, but the percentage who had received an influenza vaccination in each of these groups was lower than the percentage among Asian adults. Vaccination coverage increased significantly with each increasing level of family income for White adults only.

**Source**: National Center for Health Statistics, National Health Interview Survey, 2021 data. https://www.cdc.gov/nchs/nhis.htm

